# Enhancing melanoma therapy with hydrogel microneedles

**DOI:** 10.3389/fonc.2025.1590534

**Published:** 2025-04-17

**Authors:** Lanqi Zhu, Guanlin Qiao, Huiyang Gao, Aowei Jiang, Linan Zhang, Xiaobing Wang

**Affiliations:** ^1^ The First Clinical Medical College, Shanxi Medical University, Taiyuan, Shanxi, China; ^2^ Department of Plastic and Reconstructive Surgery, First Hospital of Shanxi Medical University, Taiyuan, Shanxi, China

**Keywords:** hydrogel microneedles, melanoma, cancer therapy, drug delivery systems, controlled release

## Abstract

Melanoma is highly invasive and resistant to conventional treatments, accounting for nearly 75% of skin cancer-related deaths globally. Traditional therapies, such as chemotherapy and immunotherapy, often exhibit limited efficacy and are associated with significant side effects due to systemic drug exposure. Microneedles (MNs), as an emerging drug delivery system, offer multiple advantages, including safety, painlessness, minimal invasiveness, and controlled drug release. Among these, hydrogel microneedles (HMNs) stand out due to their extracellular matrix-like structure and swelling-induced continuous hydrogel channels, which enable the direct delivery of therapeutic agents into the tumor microenvironment (TME). This approach enhances drug bioavailability while reducing systemic toxicity, establishing HMNs as a promising platform for melanoma treatment. This review highlights recent advancements in HMNs for melanoma therapy, focusing on their applications in biomarker extraction for early diagnosis and their role in supporting multimodal treatment strategies, such as chemotherapy, immunotherapy, phototherapy, targeted therapy, and combination therapy. Furthermore, the current matrix materials and fabrication techniques for HMNs are discussed. Finally, the limitations of HMNs in melanoma treatment are critically analyzed, and recommendations for future research and development are provided.

## 1 Introduction

Melanoma, a malignant tumor originating from melanocytes, has shown a globally increasing incidence (1, 2). As one of the deadliest forms of skin cancer, it accounts for over 60,000 deaths annually worldwide (3). Although conventional treatments—including surgical resection, immunotherapy, radiotherapy, chemotherapy, and targeted therapy (4)—have demonstrated some efficacy, their therapeutic potential is often hindered by the highly invasive and drug-resistant nature of the disease (5). Achieving complete remission frequently requires high drug doses, which not only increases treatment complexity but also elevates the risk of severe side effects (6). These limitations underscore the urgent need for innovative approaches to improve therapeutic outcomes through safer and more efficient drug delivery systems.

Transdermal Drug Delivery Systems (TDDS) represent a non-invasive strategy for delivering drugs directly into dermal or subdermal tissues via the skin (7). By bypassing the gastrointestinal tract, TDDS avoids first-pass metabolism, thereby enhancing drug bioavailability at targeted sites. Additionally, TDDS offers a convenient and painless delivery method (8). Among various TDDS techniques, microneedles have emerged as a promising platform due to their ability to penetrate the epidermis and create microchannels while avoiding contact with capillaries and nerves (9). In cancer therapy, microneedles enable localized delivery of anticancer immunogens, adjuvants, and other therapeutic agents, thereby increasing drug concentrations at tumor sites and reducing toxicity to healthy tissues (10). Recent advancements in MN-based systems have highlighted their potential to enhance cancer treatment outcomes. For instance, Song et al. (11) developed a dissolvable microneedle system for delivering nano-photosensitizers. Under near-infrared (NIR) irradiation, this system effectively eliminated residual breast cancer cells, induced immunogenic cell death, reduced tumor recurrence and metastasis, and enhanced systemic immune memory when combined with aPD-L1 therapy. Similarly, Liu et al. (12) designed a microwave-responsive microneedle patch incorporating magnetic biomimetic metal-organic frameworks and platelets. This system activates platelets, releases therapeutic agents, and promotes deeper drug penetration, showing promise for treating deep-seated tumors such as breast cancer and melanoma. Furthermore, D’Amico et al. (13) utilized microneedle patches to deliver cancer vaccines composed of peptide-coated conditionally replicating adenoviruses and tumor-associated antigens, achieving complete tumor rejection in melanoma models.

**Figure 1:**
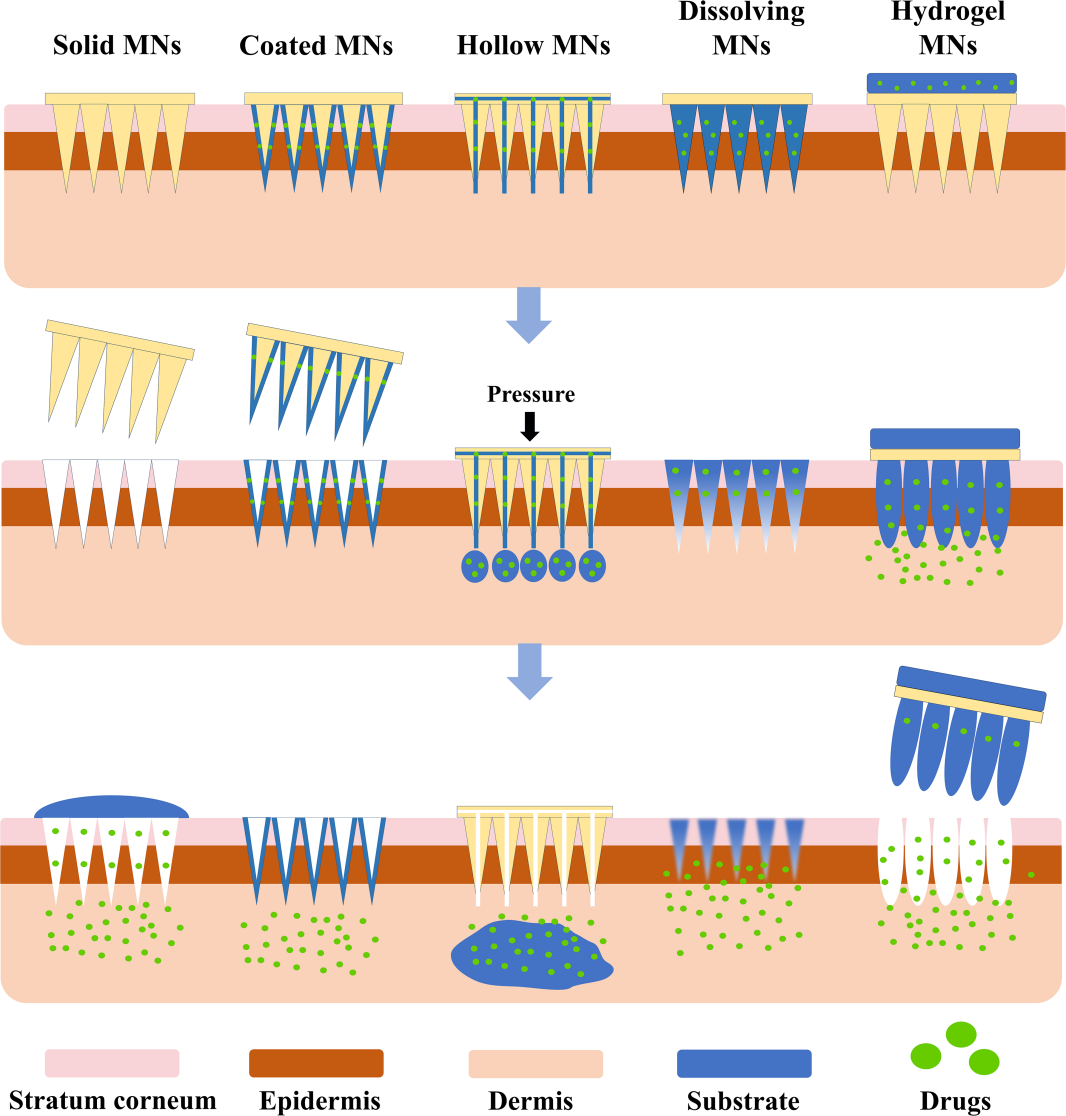
Structure and drug release mechanisms of various MN types.

Microneedles (MNs) can be categorized into five types based on their drug delivery mechanisms: solid, coated, hollow, dissolving, and hydrogel microneedles (**Figure 1**) (14). **Table 1** summarizes the materials, advantages, and disadvantages of these MN types. Among them, hydrogel MNs, made from hydrogel-based materials, mimic the extracellular matrix and exhibit excellent biocompatibility (15). Upon insertion into the skin, their hydrophilic properties allow them to swell and form continuous hydrogel channels, which are particularly advantageous for biomedical applications such as interstitial fluid (ISF) extraction (16). These channels facilitate drug diffusion, ensuring stable and sustained therapeutic effects (17).

**Table 1:** Materials, advantages, and disadvantages of different MN types.

MN Type	Materials	Advantages	Disadvantages	Ref.
Solid MN	Metallic materials or inorganic non-metallic substances (e.g., silicon and ceramics)	Strong mechanical strength; simple structure	Non-degradable; residues remain in the skin	(18–20)
Coated MN	Metal or silicon materials	Facilitates rapid drug delivery to the skin	Limited coating thickness restricts high-dose drug delivery	(21, 22)
Hollow MN	Nickel, stainless steel, silicon, ceramics	Efficiently delivers large volumes of drug solutions; avoids drug wastage	Needle tip apertures are prone to blockage by skin tissues	(23–25)
Dissolving MN	Biocompatible and biodegradable polymers	Convenient; no residue risk	Poor penetration strength	(26, 27)
Hydrogel MN	Polymers	Superior biocompatibility; high drug-loading capacity	Mechanical strength limitations	(15, 28)

**Figure 2:**
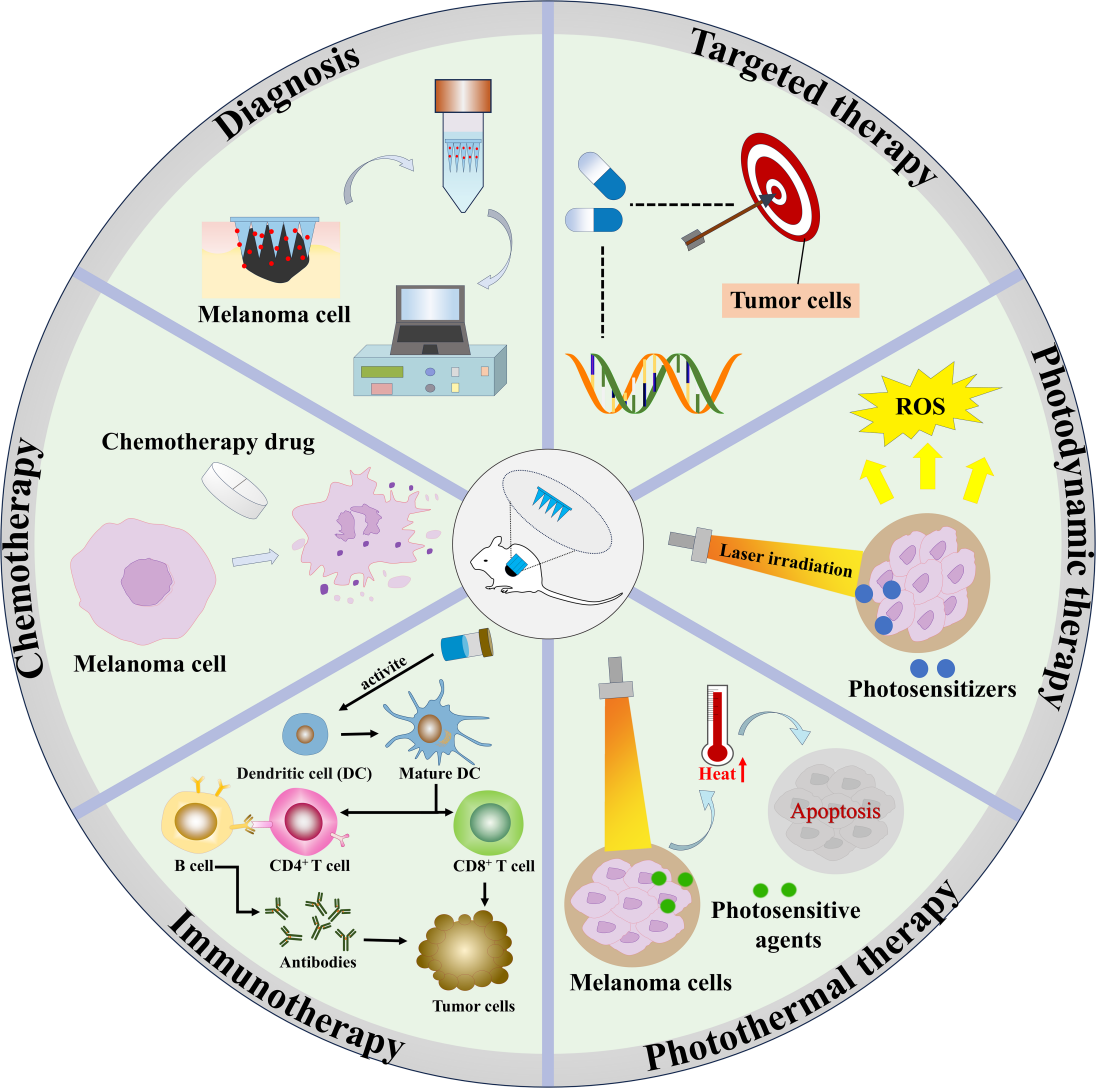
The applications of HMNs in melanoma diagnosis and therapy.

HMNs have demonstrated considerable potential in melanoma treatment due to their efficient drug delivery capabilities, non-invasive nature, and high biocompatibility. These properties enable hydrogel microneedles to address the challenge of low bioavailability while minimizing systemic drug exposure (29). This review explores the applications of HMNs in melanoma diagnosis and therapy, including chemotherapy, targeted therapy, immunotherapy, photothermal therapy (PTT), photodynamic therapy (PDT), and combination therapies (**Figure 2**). Additionally, it provides an overview of the materials and fabrication techniques employed in HMN production, examines their strengths and limitations in the context of melanoma treatment, and outlines future directions for their development. Ultimately, this review aims to establish a foundation for further research on hydrogel microneedle-based drug delivery systems for melanoma, facilitating their clinical translation and contributing to improved patient outcomes.

## 2 Design of HMNs

HMNs exhibit exceptional water absorption and swelling properties, enabling the gradual release of drugs after skin penetration, thus facilitating sustained therapy (30). Compared to traditional microneedles, HMNs demonstrate superior biocompatibility and allow for tunable drug release rates by adjusting the crosslinking density, which directly influences the hydrogel’s network structure (31).

### 2.1 Matrix materials

The performance of HMNs is profoundly affected by the selection of matrix materials, which can be broadly categorized into natural and synthetic hydrogels (32). **Table 2** summarizes the commonly used materials for HMNs. Natural hydrogels, including silk fibroin (SF) (33), methacrylated hyaluronic acid (MeHA) (34), gelatin methacryloyl (GelMA) (35), sodium alginate (SA) (36), and chitosan (CS) (37), are frequently employed due to their excellent biocompatibility. Synthetic hydrogels, such as poly (vinyl alcohol) (PVA) (38) and poly (methyl vinyl ether-co-maleic acid) (PMVE/MA) (39), provide enhanced mechanical strength and tunable physicochemical properties, making them particularly suited for sophisticated and complex drug delivery systems (40).

**Table 2:** Base materials, loaded drug components, and features of different HMNs.

Base Material	Loaded Drug Components	Features	Ref.
Silk fibroin methacrylate	α-MSH	Protects melanocytes in vitiligo patients and restores skin pigmentation	(41)
MeHA	Zn-MOF	Exhibits antibacterial properties, accelerates skin regeneration, and promotes neovascularization	(42)
SF	Riboflavin	Enables precise 3D printing in low-concentration aqueous solutions	(33)
MeHA	methotrexate	Prolongs the retention time of drugs within the skin	(34)
GelMA	cerium dioxide@ taurine nanoparticles	Provides antioxidative, anti-inflammatory, and anti-aging effects; promotes diabetic wound healing	(35)
GelMA	Oncolytic NDV	Facilitates cancer immunotherapy; selectively kills liver cancer cells	(43)
Ca²⁺ cross-linked alginate	EAA, tranexamic acid	Enhances drug penetration efficiency; suitable for acidic drugs	(44)
CS	5-FU PEGylated liposomes	Improves penetration efficiency and demonstrates efficacy in treating early-stage skin lesions	(45)
CS	Rifampicin	Enables temperature-sensitive drug release and accelerates wound healing	(37)
PVA and SA	Composite nanoyzyme	Rapidly extracts ISF and assists in the colorimetric detection of glutathione	(38)
PVA	MSC derived exosomes	Promotes wound repair and tissue regeneration	(46)
PMVE/MA	Caffeine	Enhances skin permeability and improves transdermal drug delivery efficiency	(39)
PEG and PMVE/MA	Acyclovir	Improves skin penetration efficiency and enhances transdermal delivery	(47)
DA-HA, PEDOT: PSS	Not used	Exhibits excellent electrical sensitivity and enables pH sensing	(48)
MeHA and LAP	OVA	Provides prolonged immune effects and dendritic cell activation	(49)

Natural hydrogels exhibit low immunogenicity, excellent cellular compatibility, and biodegradability. Their functional groups enable specific responses targeting particular cells or tissues (50, 51). For instance, silk fibroin methacrylate HMNs have been utilized to deliver α-melanocyte-stimulating hormone (α-MSH), effectively improving melanocyte dysfunction in vitiligo patients (41). Yao et al. (42) developed a wound dressing based on a Zn-MOF (metal–organic framework) encapsulated MeHA microneedle array. This dressing demonstrated antibacterial activity, while the hydrolysis of MeHA promoted tissue repair, epithelial regeneration, and angiogenesis. Similarly, GelMA microneedles have been employed as a delivery platform for the alginateoncolytic Newcastle disease virus (NDV), offering a novel approach in cancer immunotherapy (43). Zhou et al. (44) designed calcium ion (Ca²⁺)-crosslinked alginate swellable microneedles, significantly enhancing the transdermal delivery efficiency of acidic drugs, such as 3-O-ethyl ascorbic acid (EAA), while ensuring stable release over a 16-hour period. Additionally, Suriyaamporn et al. (45) developed a chemotherapy patch based on chitosan HMNs loaded with 5-fluorouracil (5-FU) liposomes. This innovative patch not only promotes wound healing and exhibits anti-cancer activity but also demonstrates no significant toxicity to normal cells.

While natural hydrogels are highly biocompatible, they often lack optimal mechanical properties (52). In contrast, synthetic hydrogels can achieve superior mechanical strength and rigidity by modifying polymer structures or adjusting crosslinking degrees (53). For instance, researchers crosslinked PVA and (polyvinylpyrrolidone) PVP with glutaraldehyde at 95°C, incorporating a spiral geometric design to enhance skin contact area, reduce insertion force, and improve stability. Ultraviolet crosslinking further reinforced compressive resistance, resulting in optimized PVA/PVP/HA (hyaluronic acid) microneedles with a fracture force of 0.13 N, far exceeding the 0.058 N required for skin penetration. Additionally, applying micro-vibration at 50 Hz and 100 Hz during insertion reduced skin resistance and minimized fracture risk (54). Similarly, Li et al. (55) developed crosslinked hydrogels using PVA, PVP, and poly(ethylene glycol) diacid (PEGdiacid), optimizing conditions with a PEGdiacid concentration of 2.25% w/w and crosslinking at 150°C for 20 minutes. A 14×14 conical mold was employed to enhance stress distribution, effectively reducing compressive deformation. The optimized microneedles demonstrated a height reduction of only 7.67% under 32 N pressure and achieved insertion depths of 508–522 μm, successfully penetrating the stratum corneum while maintaining structural integrity. These microneedles exhibited excellent mechanical properties and insertion performance, providing robust support for transdermal drug delivery. Furthermore, synthetic hydrogels exhibit enhanced water absorption capabilities compared to their natural counterparts (50). For example, Zhang et al. (46) developed bioinspired adaptive retention microneedles by combining tunable PVA HMNs with mesenchymal stem cell (MSC)-exosomes for transdermal drug delivery in diabetic wound treatment. By employing 3D printing and template replication techniques, the mechanical properties of the PVA hydrogel were modulated using sulfate ions to increase strength, while nitrate ions softened its structure, allowing for tissue adaptation and controlled exosome release. In another study, Al-Badry et al. (47) developed a microneedle array composed of a copolymer of poly(ethylene glycol) (PEG) and PMVE/MA for the transdermal delivery of acyclovir. This microneedle array achieved a 75.56% release of acyclovir within 24 hours, with a transdermal absorption rate 39 times higher than the control. This approach effectively reduces the adverse effects associated with oral administration and enhances patient compliance.

Advancements in medical technology have highlighted the limitations of traditional materials in drug delivery and personalized therapy. To address these challenges and expand their applications, researchers have explored the integration of novel materials into microneedle fabrication. The incorporation of materials such as Laponite, conductive materials, and light-responsive materials has significantly enhanced the overall performance of HMNs. Odinotski et al. (48) developed a HMN biosensor for real-time pH measurement by integrating dopamine(DA)-conjugated HA hydrogel with poly(3,4-ethylenedioxythiophene): polystyrene sulfonate. The HA hydrogel efficiently collected ISF and enabled precise pH measurement, while PEDOT: PSS improved the microneedles’ electrical conductivity and mechanical strength. Experimental results demonstrated that this HMN biosensor achieved 93% accuracy in pH solution testing, showcasing its potential for wearable sensors and health monitoring applications. Zheng et al. (49) designed a composite hydrogel based on photo-crosslinked MeHA and Laponite (LAP). The addition of LAP significantly enhanced the microneedles’ mechanical strength and extended the release duration of antigens, such as ovalbumin (OVA). This improvement promoted better antigen uptake and presentation efficiency, highlighting the composite’s potential in vaccination applications. Therefore, the selection of materials for HMN fabrication is pivotal, as it directly influences drug delivery precision, safety, and therapeutic efficacy.

### 2.2 Formation mechanism

The gelation of hydrogels occurs through various mechanisms, with the most common being physical cross-linking and chemical cross-linking (56). These mechanisms synergistically form polymer networks with hydrophilic and water-swelling properties (57). The porous structure of these networks facilitates drug encapsulation, minimizing drug loss and enhancing therapeutic efficacy. Additionally, the tunability of the polymers allows for the optimization of microneedles’ physical and chemical properties, enabling controlled drug delivery (58).

#### 2.2.1 Physical cross-linking

Physical cross-linking relies on non-covalent interactions—such as ionic interactions, hydrogen bonding, and hydrophobic interactions—to form hydrogel networks (59). These hydrogels avoid the use of toxic organic solvents, exhibit high biocompatibility, and are reversible since the interactions between polymer chains do not involve covalent bonds (60). Furthermore, physically cross-linked hydrogels can adjust their mechanical properties in response to environmental conditions, such as temperature and pH (61). However, their mechanical performance is generally lower, and the reversible nature of the cross-linking can lead to reduced stability under physiological conditions (62).

#### 2.2.2 Chemical cross-linking

Chemical cross-linking, in contrast, is an irreversible process that involves the formation of covalent bonds between polymer chains. These bonds are typically created through free radical polymerization, functional group reactions, or high-energy radiation-induced reactions, resulting in a stable three-dimensional network (63, 64). Chemically cross-linked hydrogels exhibit superior mechanical strength and elastic modulus, making them ideal for applications requiring high stability and durability (65). However, chemical cross-linking often requires toxic cross-linking agents, and the process may generate harmful byproducts, raising concerns about environmental and human health risks (66). To conclude, by rationally combining or independently utilizing physical and chemical cross-linking methods tailored to specific medical needs, hydrogels with enhanced performance can be designed, achieving an optimal balance of biocompatibility, mechanical strength, and stability.

### 2.3 Geometric parameters

When designing HMNs, several geometric parameters must be carefully considered, including microneedle shape, length, tip sharpness, and density (67, 68). These factors are critical to the clinical applicability, manufacturing feasibility, and drug-loading capacity of microneedles, directly influencing their performance across various applications (69). Common microneedle shapes include pyramid-shaped (70), cone-shaped (71), claw-inspired (72), shark tooth-shaped (73), and pagoda-shaped bioinspired structures (74). Studies have shown that increasing the number of edges in the cross-sectional shape of the microneedle tip (e.g., square, hexagonal, and octagonal) enhances swelling capacity and drug release rates. However, simpler cross-sectional shapes with fewer edges allow for more thorough drug release (69). Microneedle length is another critical parameter, particularly in relation to pain perception (75). In a double-blind randomized controlled trial, Gill et al. observed that increasing microneedle length from 480 μm to 1450 μm resulted in a 7-fold increase in pain perception, while variations in width, thickness, and tip angles had no significant effect. Additionally, increasing the number of microneedles tenfold (from 5 to 50) caused only a 2.5-fold increase in pain, highlighting microneedle length as the dominant factor influencing pain perception. Tip sharpness also plays a significant role in insertion efficiency and pain reduction. High-resolution imaging studies have revealed that sharper microneedle tips significantly lower insertion force compared to variations in tip angles, thereby minimizing pain (76). Microneedle density has a notable impact on skin recovery. Low-density microneedle arrays (e.g., 121 MNs/cm²) result in less tissue damage and faster recovery, whereas high-density arrays (e.g., 361 MNs/cm²) can cause greater tissue damage and slower recovery. Excessively high-density arrays may compromise the long-term recovery of the skin barrier and induce the “bed of nails” effect, further exacerbating tissue damage and delaying healing (77). In conclusion, optimizing geometric parameters such as shorter microneedle lengths, enhanced tip sharpness, and appropriately spaced microneedle arrays is essential for achieving minimally invasive, painless, and effective drug delivery.

### 2.4 Preparation methods

The most common preparation methods for HMNs are micromolding technology (78) and 3D printing-based methods (79). Each method offers unique advantages and is suitable for different production requirements.

Micromolding technology is widely used for HMN fabrication due to its reusable micromolds and high-efficiency mass production capabilities (80). Micromolds can be fabricated using a variety of techniques, including thermoforming, injection molding, laser drilling, and polymer casting, to address diverse precision and manufacturing requirements (81, 82). Among these, polydimethylsiloxane (PDMS) is the most commonly used micromold material (75). Its flexibility and hydrophobicity make it an optimal choice for microneedle fabrication (83). PDMS microneedle templates are typically prepared using a replica molding process. In this method, solid MNs—mechanically rigid materials—are used as the master mold, onto which a mixture of PDMS prepolymer and curing agent is cast. After curing, the PDMS template is formed. The polymer solution is then filled into the template, and vacuum or centrifugal forces are applied to ensure uniform distribution. Microneedle formation is completed through drying or photopolymerization, followed by demolding (84, 85). This process is simple, efficient, and cost-effective due to the reusability of PDMS templates, making it ideal for scalable HMN production.

As an additive manufacturing technique, 3D printing constructs three-dimensional structures by depositing materials layer by layer. Its high precision and rapid prototyping capabilities have made it an innovative approach for microneedle fabrication (86). Among the various 3D printing methods, photopolymerization is the most commonly employed technique. Photopolymerization selectively cures photosensitive materials using lasers or light sources to build microneedle structures layer by layer (87). Common photopolymerization methods include Digital Light Processing and Stereo Lithography Apparatus, which enable the high-precision fabrication of HMNs with excellent tensile properties and biocompatibility (88). Compared to traditional micromolding, 3D printing not only allows for the direct fabrication of complex microneedle geometries but also provides precise control over size, shape, and structure, enabling highly customizable designs (89). Additionally, 3D printing can be used to produce microneedle negative molds, which, when combined with replica molding processes, further optimize production workflows. This integration significantly enhances both the flexibility and efficiency of microneedle manufacturing (79).

## 3 HMNs for melanoma diagnosis

**Figure 3:**
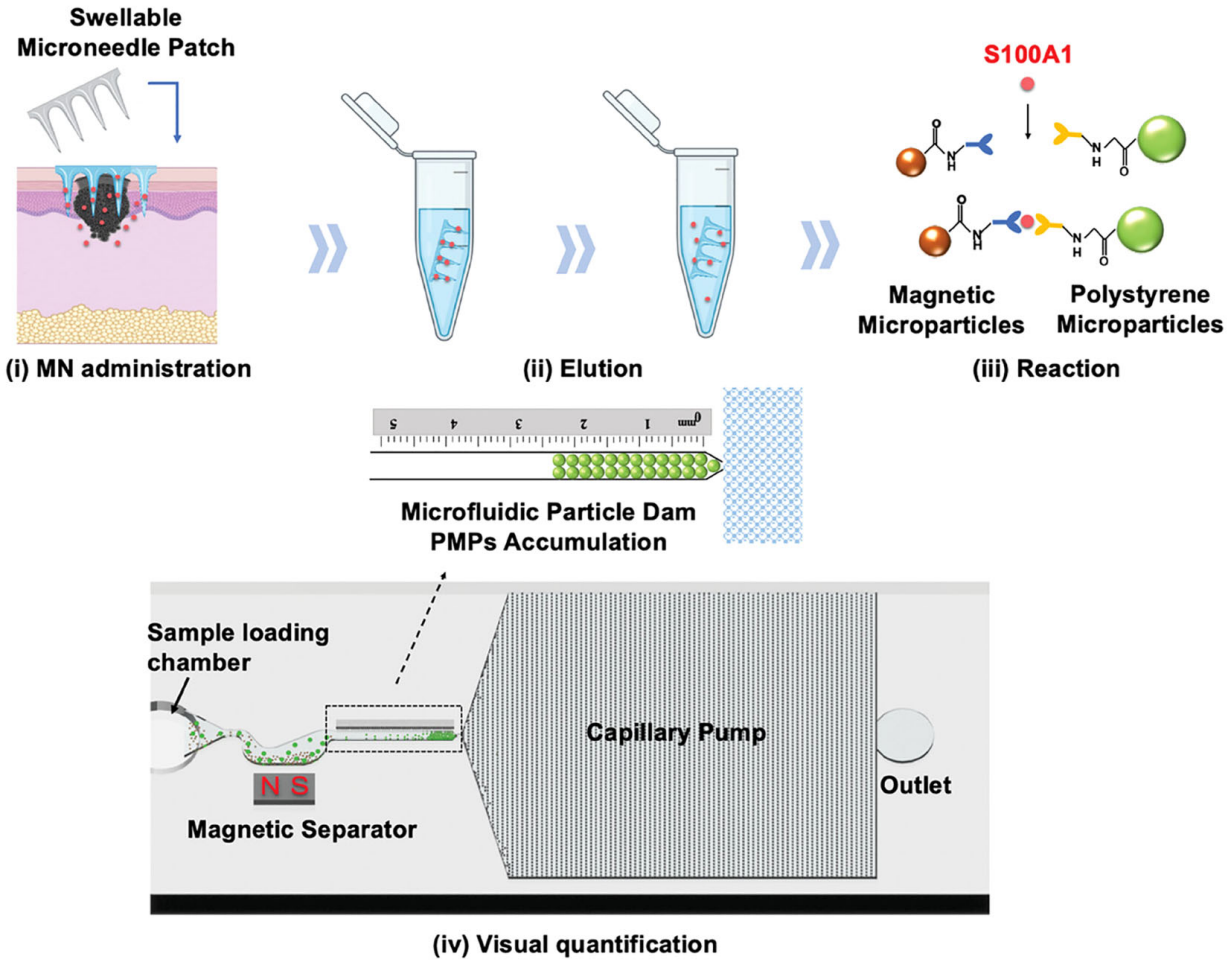
Schematic illustration of the HMN patch for melanoma diagnosis. (i) ISF is extracted using the microneedle patch, (ii) eluted into a centrifuge tube, (iii) where S100A1 binds to MMPs and PMPs, and (iv) its concentration is quantified by measuring PMP accumulation. Copyright permission from Wang et al. (97), *Advanced Science*, 2024.

The early clinical manifestations of melanoma closely resemble those of benign pigmented nevi, which limits the diagnostic accuracy of dermoscopy as a non-invasive tool (90). Due to the rapid metastatic progression of melanoma, most patients are diagnosed at advanced stages, leading to poor prognosis (91). This underscores the urgent need for novel biomarkers to improve diagnostic accuracy. Melanoma-associated biomarkers, such as the S100 protein family, miRNA, and exosomes, have been identified in blood (92). While microneedles have been utilized to extract biomarkers like S100B, tyrosinase, and lactate from skin ISF for diagnostic purposes, fluorescence- or colorimetric-based detection methods often lack quantification capabilities, negatively affecting diagnostic specificity (93). Studies have shown that serum S100A1 expression levels are significantly elevated in melanoma patients compared to non-melanoma individuals (94). However, S100A1 overexpression is also associated with other diseases, including breast cancer, cardiovascular diseases, and ovarian cancer, which limits its specificity for melanoma diagnosis (95, 96). To address this challenge, Wang et al. (97) developed a HMN patch based on MeHA for the early diagnosis of melanoma. This microneedle patch extracts S100A1 from skin ISF and utilizes antibody-conjugated magnetic microparticles (MMPs) and polystyrene microparticles (PMPs) for the quantitative measurement of S100A1 concentration, achieving an impressive detection limit of 18.7 ng/mL. Animal experiments demonstrated that this method exhibits higher sensitivity at lower concentrations compared to conventional serum-based detection methods. To enhance usability, the researchers incorporated microfluidic technology, which made the detection results more intuitive and easier to interpret. The integration of microneedles with a microfluidic system streamlined the operational workflow, making it suitable for home use or routine skin examinations (**Figure 3**). However, despite its high sensitivity, this method is currently limited to detecting S100A1 alone, excluding other potential melanoma biomarkers, which restricts its broader applicability. Additionally, the reliance on a standard curve for quantification introduces the possibility of errors under varying conditions, potentially impacting the accuracy of measurements.

## 4 HMNs for melanoma treatment

HMNs have shown great potential as a drug delivery platform for melanoma treatment. Their unique properties, including excellent drug-controlled release, biocompatibility, and minimally invasive transdermal drug delivery, make them an attractive option for localized melanoma therapy. Compared to conventional treatments, HMNs can significantly reduce systemic toxicity, precisely target tumor sites, enhance therapeutic efficacy, and minimize drug-related side effects. Accordingly, this section provides a comprehensive summary of the applications of HMNs in melanoma treatments, encompassing chemotherapy, immunotherapy, targeted therapy, phototherapy, and synergistic therapy (see **Table 3**). This overview aims to serve as a valuable reference for developing related therapeutic strategies.

**Table 3:** Applications of HMNs in melanoma treatment.

Microneedle Material	Application	Therapeutic Mechanism	Drug Type	Therapeutic Advantages	Challenges and Limitations	Ref.
GelMA	Sustained transdermal delivery for anticancer therapy	Enhances transdermal delivery and controls drug release by modulating cross-linking density.	DOX	Enables precise control of drug release.	Validated only in animal models; lacks clinical validation.	(98)
DexMA	Continuous drug delivery for chemotherapy	Delivers drugs directly to tumor sites, achieving localized release and reducing systemic toxicity.	DOX, Tra	Reduces multidrug resistance.	Limited drug release rate and stability in long-term applications.	(99)
PVA	Localized chemotherapy	Pd nanoparticles catalyze the activation of prodrugs, enabling localized drug activation for tumor treatment.	Alloc-DOX	Reduces drug- induced side effects on healthy tissues.	Catalyst- induced leakage; inability to address broad metastases.	(100)
Crosslinked HA	Cancer immunotherapy; immune response monitoring	Immunostimulatory molecules activate dendritic cells and macrophages through binding to the TLR9 receptor.	CpG-ODNs	Combines treatment with monitoring of immune response biomarkers	Variability in individual immune responses.	(101)
GelMA-β-CD	Targeted drug delivery	Delivers drugs directly to the tumor region via microneedle arrays.	Curcumin	Significantly enhances stability of curcumin for water-insoluble drugs.	Limited applicability for water-insoluble drugs.	(102)
MeHA	Combines PTT with immunotherapy	Delivery of melanin nanoparticles and immunoadjuvants, activation of immune response via NIR irradiation, and enhancement of T cell- mediated tumor targeting.	Tumor lysate, melanin nanoparticles, GM-CSF	Amplifies photothermal and immune synergistic effects.	Limited photothermal depth due to tumor location.	(103)
HA	Enhances PDT	Delivers a self- oxygenating and GSH depleting nanoplatform to enhance the effectiveness of PDT	CZCH	Reduction of phototoxicity; enhancement of PDT efficacy.	Complex technology with limited therapeutic depth.	(104)
HEMA and NVP copolymer	Localized delivery of CO to enhance chemosensitivity in tumor cells	Photocatalytic conversion of CO2 to CO, enhancing tumor cell sensitivity to chemotherapeutic agents.	Cisplatin	Amplifies therapeutic efficacy.	Relies on photocatalysts.	(105)
PVA and PVP	Transdermal delivery for anticancer drug administration	Facilitates drug penetration through the skin, achieving controlled release to enhance anticancer effects.	AZA, MAT	Cost-effective; improves the stability of MAT.	Drug degradation and partial instability.	(106)
HA, PVA, and PVP	Melanoma ablation and postoperative skin regeneration	Combines PTT, PDT, and immune activation to eliminate tumors, while leveraging the hydrogel matrix to accelerate tissue repair.	CCa@TF/Ce6	Integrates tumor treatment with skin regeneration.	Limited treatment depth; requires further safety validation for long-term use.	(107)
HAT	PDT for melanoma	Activates photosensitizers under laser irradiation to generate reactive oxygen species and destroy tumor cells.	TMPyP	Tunable mechanical strength.	Complex preparation process.	(108)
HA	Noninvasive monitoring of melanoma immunotherapy	Enables collection of ISF biomarkers for detecting immune responses.	Not used	Provides real-time, noninvasive assessment of immunotherapy efficacy.	Limited ISF volume collected (<3 μL);	(109)

### 4.1 Microneedles for chemotherapy

Chemotherapy remains a cornerstone in anti-cancer treatments; however, its therapeutic efficacy is significantly hindered by low bioavailability, insufficient drug accumulation in tumor regions, systemic toxicity, and the development of chemoresistance (110, 111). Commonly used chemotherapeutic drugs for melanoma, such as Paclitaxel, Doxorubicin (DOX), and Cisplatin, are associated with systemic toxicity and adverse side effects (112, 113). Delivering chemotherapeutic agents directly to tumor sites via MNs represents a safer and more effective therapeutic strategy.

**Figure 4:**
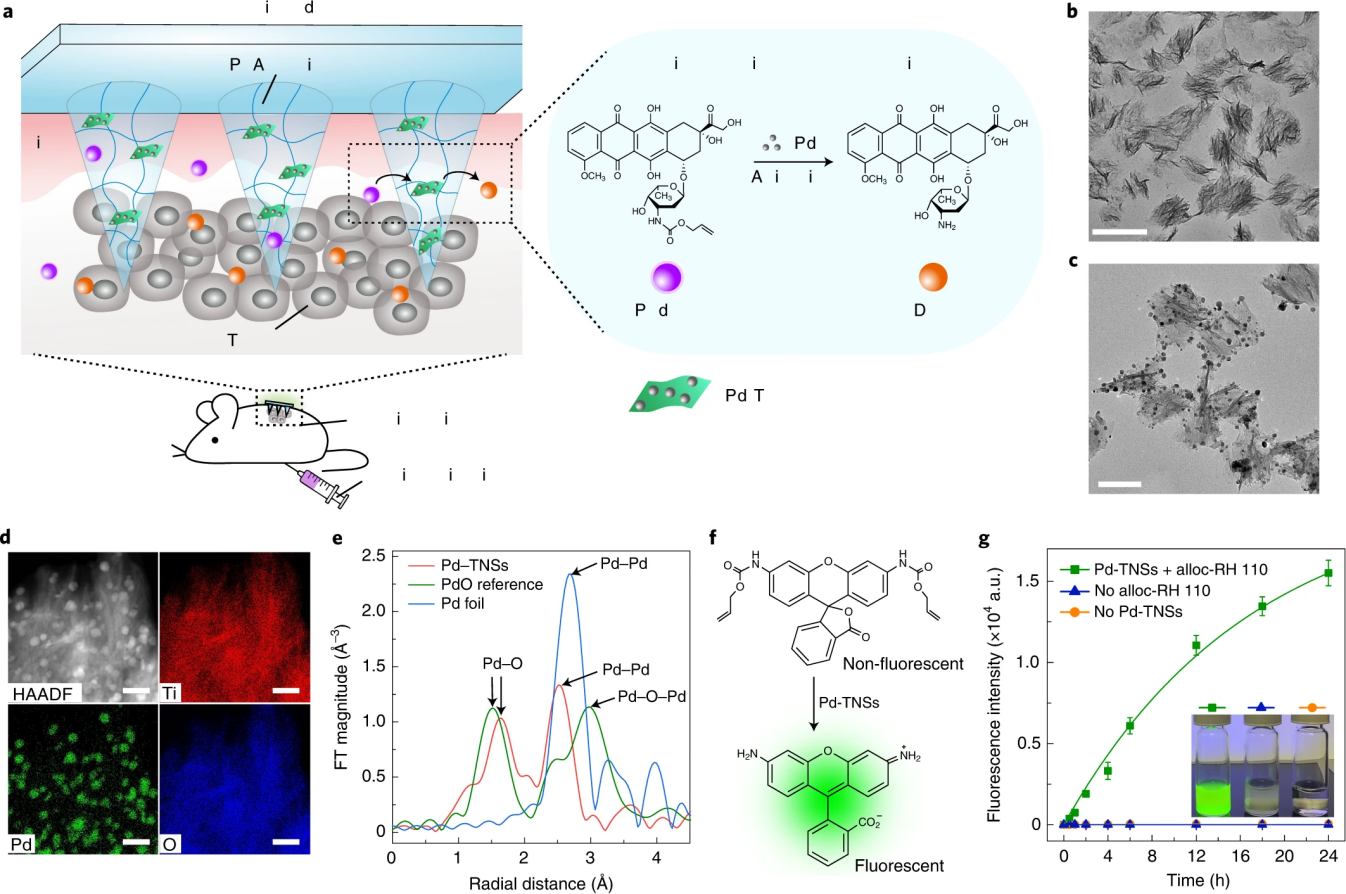
Schematic design of the MN patch for chemotherapy and characterization of Pd-TNSs.**(a)** Schematic illustration of bioorthogonal catalysis; **(b)** transmission electron microscope images of TNSs and **(c)** Pd-TNSs; **(d)** elemental mapping analysis of Pd-TNSs; **(e)** structural characterization of palladium in Pd-TNSs through extended X-ray absorption fine structure (EXAFS); **(f)** schematic representation of the decaging process; **(g)** fluorescence intensity measurements for monitoring drug activation and release. Copyright permission from Chen et al. (100), *Nature Nanotechnology*, 2021.

In drug delivery systems, one of the critical challenges is achieving an optimal drug release rate, as release tends to be either too fast or too slow. To address this, Luo et al. (98) developed a microneedle patch based on GelMA for the sustained and controlled release of the anticancer drug DOX, facilitating effective melanoma treatment. Experimental results indicated that GelMA microneedles loaded with DOX could efficiently penetrate mouse skin. By tuning ultraviolet exposure time and intensity, the crosslinking density of the microneedles could be adjusted, allowing control over their mechanical properties and drug release behavior. Specifically, low crosslinking density resulted in rapid swelling and accelerated drug release, whereas high crosslinking density slowed the release process. Despite its widespread use, the efficacy of DOX is often reduced by P-glycoprotein (P-gp)-mediated multidrug resistance. To overcome this limitation and enhance therapeutic outcomes, Huang et al. (99) developed a novel microneedle system using Dextran Methacrylate (DexMA) hydrogel, which co-delivers DOX and the resistance-reversal drug Trametinib (Tra). This dual-drug system effectively inhibits the efflux of DOX and reverses P-gp-mediated drug resistance. Experimental results demonstrated that the synergistic effect of DOX and Tra significantly improved local anti-melanoma efficacy while minimizing systemic toxicity and side effects. Although microneedles provide sustained drug release, the release rate may be limited by variations in drug solubility, such as the poor water solubility of Tra. Furthermore, the long-term toxicity of DOX to healthy tissues has not been fully evaluated. To address this limitation, Chen and colleagues (100) developed a bioorthogonal catalytic patch based on a PVA matrix to enhance anti-melanoma efficacy while reducing DOX-associated toxicity in healthy tissues. This microneedle array patch was loaded with a composite material consisting of palladium (Pd) nanoparticles and titanium dioxide nanosheets (TiO₂ nanosheets, TNSs), collectively referred to as Pd-TNSs. The palladium nanoparticles act as a catalyst, enabling the activation of the prodrug N-allyloxycarbonyl-doxorubicin (alloc-DOX) via a decaging process, which results in the release of the active drug, DOX (**Figure 4**). Notably, this reaction occurs exclusively within the specific catalytic environment of the tumor site, thereby minimizing off-target reactions *in vivo*. In a mouse melanoma model, the combination of alloc-DOX and the microneedle array patch demonstrated superior tumor growth inhibition compared to the group treated with DOX alone. Importantly, alloc-DOX itself exhibited minimal activity in healthy tissues, further reducing the risk of systemic toxicity. Although the methods utilized by the aforementioned research teams have demonstrated outstanding performance in animal studies and *in vitro* models, they have not yet been fully validated through large-scale clinical trials. Further research is required to assess their clinical applicability and accuracy.

### 4.2 Microneedles for immunotherapy

Immunotherapy has demonstrated high efficacy in treating melanoma by mobilizing or modulating the body’s immune system to recognize and attack cancer cells, thereby effectively controlling or eliminating the disease (114). Emerging immunotherapy technologies, such as polymer nanoparticles (115) and engineered immune cells (116), have shown great promise in cancer treatment. These technologies primarily facilitate the delivery of immunomodulators or create immunogenic microenvironments at tumor sites to activate immune cells.

**Figure 5:**
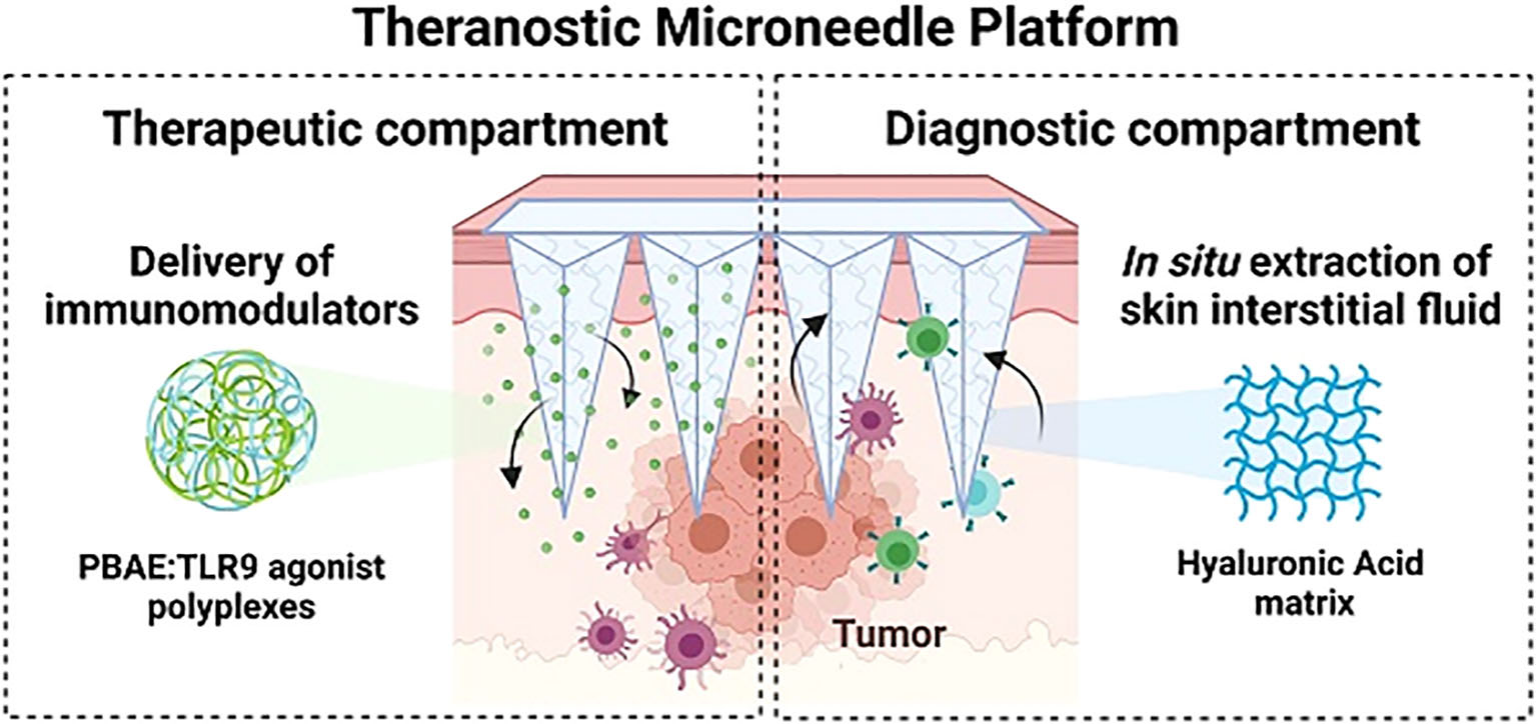
Schematic representation of the microneedle platform designed for CpG-ODN delivery and concurrent extraction of ISF. Copyright permission from Dosta et al. (101), *Theranostics*, 2023.

Cytosine-phosphate-guanine oligodeoxynucleotides (CpG-ODNs) are innate immune-activating molecules that bind to toll-like receptors on immune cell surfaces, triggering antitumor immune responses (117, 118). However, as highly water-soluble single-stranded DNA molecules, CpG-ODNs are prone to nuclease-mediated degradation, which reduces their therapeutic efficacy (119). Protecting CpG-ODNs from degradation, improving their stability, and addressing the inefficiency and dose variability associated with traditional delivery methods remain major challenges. To overcome these limitations, Dosta et al. (101) encapsulated CpG-ODNs in nanoparticles and developed a crosslinked HA microneedle platform for melanoma treatment. This platform enhances the stability of CpG-ODNs, protects them from enzymatic degradation, and facilitates precise delivery at tumor sites. CpG-ODNs activate bone marrow-derived dendritic cells and bone marrow-derived macrophages by binding to TLR9 receptors on immune cells. This activation induces cytokine release, modulates the TME and stimulates robust antitumor immune responses. Experimental studies have demonstrated that the delivery of CpG-loaded nanoparticles via this microneedle platform significantly slows tumor growth and prolongs survival in melanoma-bearing mice. Moreover, the platform incorporates theranostic capabilities by collecting ISF from the skin to analyze immune markers, enabling precise monitoring of therapeutic efficacy (**Figure 5**). This dual-function approach integrates treatment and non-invasive biomarker monitoring, improving the overall efficacy of cancer immunotherapy.

Nevertheless, traditional microneedle technology still faces limitations in sensitivity and quantification for biomarker detection after immunotherapy, particularly when monitoring low-concentration cytokines. To address these challenges, Dahis et al. (109) developed a hyaluronic acid HMN technology. This approach allows for the collection of local ISF which contains inflammatory markers closely associated with melanoma immune responses. When combined with ultrasensitive single molecule arrays, it enables precise detection of changes in key cytokine concentrations, such as IFN-β and IL-6. This system was utilized to evaluate immune responses trig-gered by a nanoparticulate stimulator of interferon genes agonist in combi-nation with thermal ablation therapy. This innovative strategy allowed for the dynamic monitoring of inflammation and tumor regression during treatment, under-scoring its potential for non-invasive, real-time monitoring. However, variability in immune system responses among patients poses a significant challenge, as the efficacy of microneedle-delivered drugs may differ between individuals. Customizing treatment strategies based on a patient’s immune status and tumor characteristics remains a critical hurdle. Future advancements in this platform to facilitate tailored treatment approaches could play a pivotal role in the development of precision medicine for cancer therapy.

### 4.3 Microneedles for targeted therapy

Targeted therapy has significantly improved survival rates in melanoma patients by selectively inhibiting specific genetic mutations or signaling pathways involved in cancer progression (120).

Curcumin, a phytochemical extracted from turmeric, exhibits potent anticancer activity by modulating various cancer-related signaling pathways and enhancing anti-melanoma effects (121). However, curcumin’s extremely limited solubility in aqueous environments severely restricts its absorption and bioavailability *in vivo* (122). Traditional oral delivery methods fail to overcome these limitations, resulting in insufficient blood concentrations and reduced therapeutic efficacy (123). To address these challenges, Zhou et al. (102) developed a melanoma-targeted microneedle delivery system composed of polymer conjugates of GelMA and β-cyclodextrin (GelMA-β-CD). β-Cyclodextrin plays a key role by forming an inclusion complex with curcumin molecules through its hydrophobic core, thereby improving curcumin’s solubility and stability. This microneedle system demonstrated high anticancer efficacy in B16F10 melanoma cells and 3D tumor models. Compared to traditional delivery methods, microneedle arrays efficiently deliver curcumin directly to melanoma sites, increasing local drug concentrations while minimizing systemic side effects.

**Figure 6:**
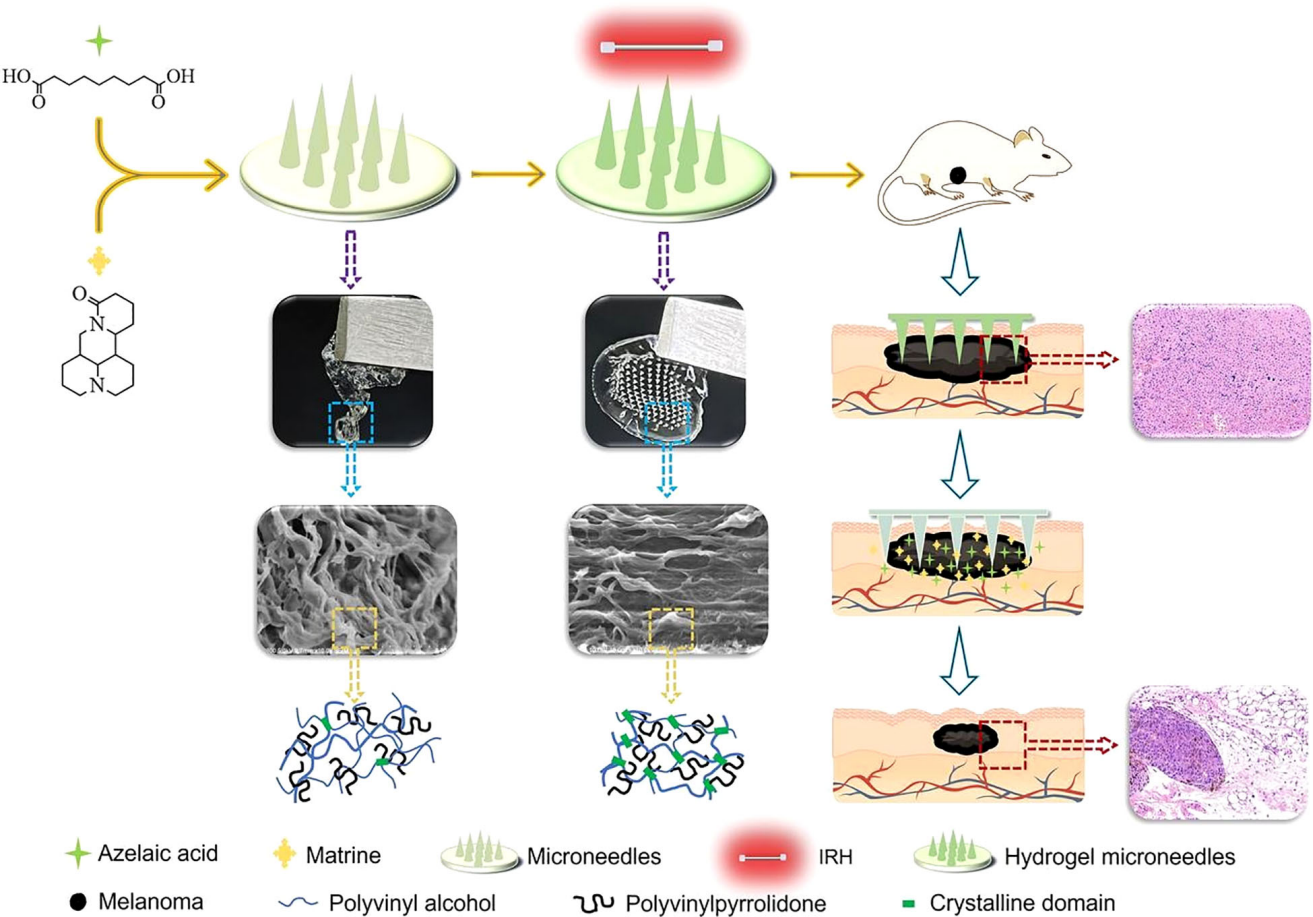
Schematic representation of HMNs for targeted melanoma therapy. Copyright permission from Xing et al. (106), *International Journal of Pharmaceutics*, 2024.

Tyrosinase is a key enzyme involved in the formation and progression of melanoma. Studies have demonstrated that tyrosinase inhibitors can suppress melanoma cell growth and induce apoptosis (124). Azelaic acid (AZA), a natural and potent tyrosinase inhibitor, has shown significant potential in inhibiting melanoma progression (125). Similarly, Matrine (MAT), the primary alkaloid extracted from the traditional Chinese herb Sophora flavescens, exhibits antitumor effects by activating the PTEN pathway. In melanoma cell lines, MAT has been shown to effectively inhibit cell proliferation and invasion, as well as induce apoptosis (126, 127). To leverage these therapeutic benefits, Xing et al. (106) developed HMNs using PVA and PVP as primary materials. The HMNs were prepared through infrared radiation heating, followed by annealing. The preparation method involved infrared irradiation at 70°C for 2 minutes, resulting in microneedles with optimal swelling properties, mechanical strength, and biocompatibility. These HMNs were loaded with AZA and MAT for targeted melanoma therapy. In a melanoma mouse model, the drug-loaded HMNs achieved a tumor inhibition rate of 90.53%, significantly outperforming the control group. The microneedles effectively released AZA and MAT simultaneously, maintaining stability during a six-month storage period without significant degradation or the generation of impurities (**Figure 6**). These findings highlight the potential of HMNs as a promising platform for targeted melanoma therapy, combining high efficacy with excellent stability and biocompatibility.

### 4.4 Microneedles for phototherapy

**Figure 7:**
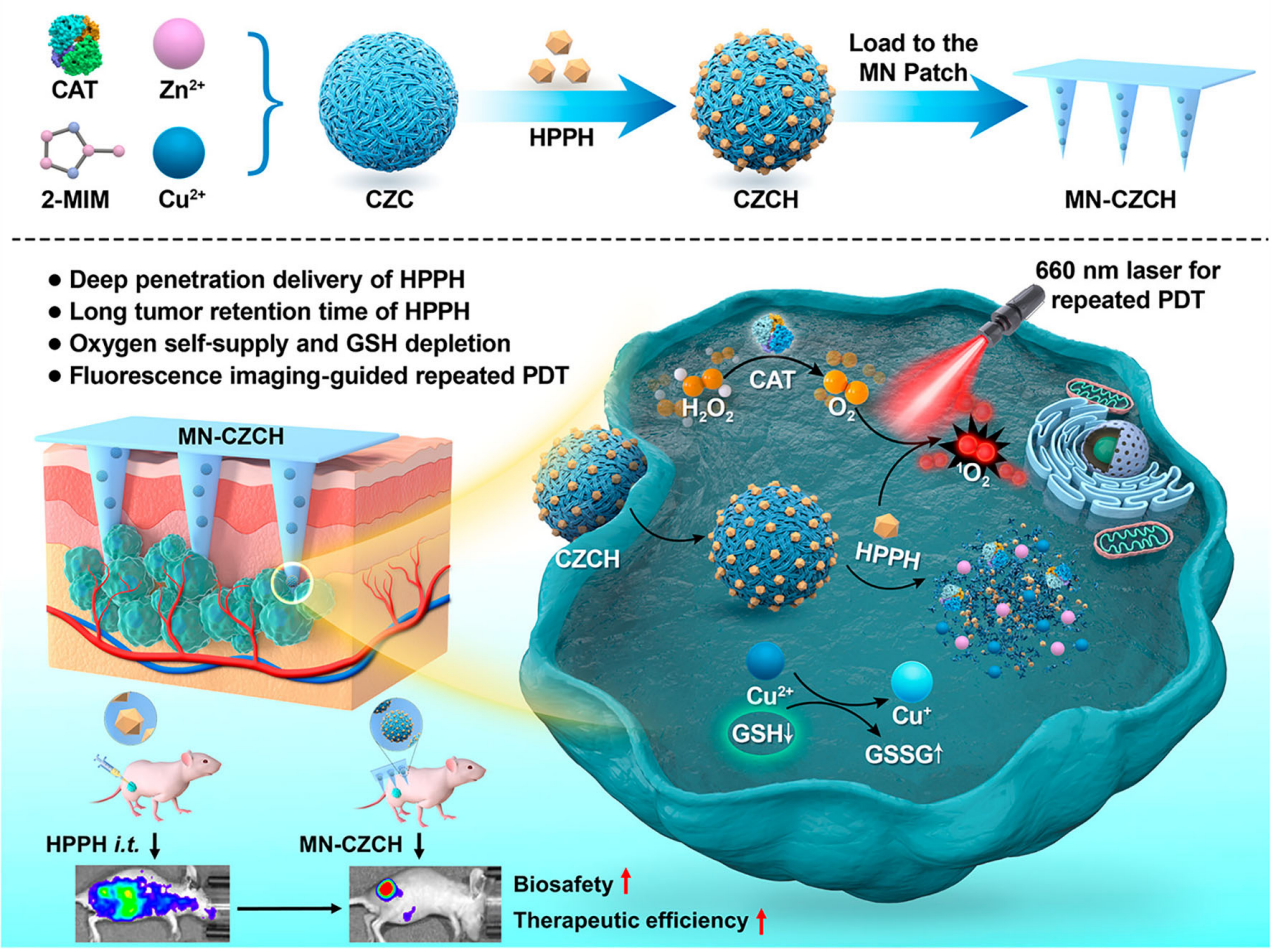
Schematic representation of HMNs for PDT in melanoma treatment. Copyright permission from Li et al. (104), *American Chemical Society*, 2022.

Traditional therapies for advanced melanoma are often associated with significant limitations, including severe side effects and inadequate drug concentrations at the tumor site. Although immunotherapy and targeted therapy have shown considerable progress, drug resistance remains a persistent challenge, leading to reduced therapeutic efficacy (128). To overcome these issues, researchers have developed phototherapy approaches such as PTT and PDT. PTT utilizes NIR light to convert light energy into heat, selectively destroying tumor cells while minimizing damage to normal cells (129). Similarly, PDT is a widely recognized non-invasive tumor treatment that involves the activation of photosensitizers to react with oxygen, producing cytotoxic reactive oxygen species (ROS) that selectively destroy tumor cells (130). However, the clinical application of PDT is hindered by several challenges, such as hypoxia in solid tumors (131, 132), skin phototoxicity caused by photosensitizers (133), and the neutralization of ROS by the elevated levels of glutathione (GSH) in cancer cells (134). To address these limitations, Li et al. (104) developed a HMN patch, termed MN-CZCH, as a drug delivery system to enhance the efficacy of PDT (**Figure 7**). Constructed from hyaluronic acid, the microneedle patch efficiently penetrates the stratum corneum to deliver the photosensitizer 2-(1-hexyloxyethyl)-2-divinylpyropheophorbic-a directly to the tumor site. The patch also incorporates a self-supplying oxygen delivery system consisting of a Cu²⁺-doped porous zeolitic imidazolate framework incorporated with catalase (CZCH). This system facilitates drug delivery while catalyzing the production of oxygen from hydrogen peroxide, thereby alleviating tumor hypoxia. Additionally, copper ions (Cu²⁺) deplete intracellular GSH enhancing the cytotoxic effects of ROS. In a melanoma mouse model, the MN-CZCH group demonstrated superior tumor inhibition under laser irradiation compared to other treatment groups, achieving a tumor growth inhibition rate of 97.7%. This performance was significantly better than that achieved with intravenous or intratumoral injection therapies. Similarly, Chi et al. (108) developed nanocomposite hydrogel microneedles with tunable mechanical strength and controllable transdermal delivery efficiency for PDT in melanoma treatment. This was achieved using an enzyme-mediated strategy. The photosensitizer tetrakis (1-methyl-4-pyridinio) porphyrin (TMPyP) was encapsulated within poly(lactic-co-glycolic acid) nanoparticles, which were then combined with a hyaluronic acid-tyramine (HAT) hydrogel. This combination enhanced the mechanical strength of the microneedles and improved drug release efficiency, providing an effective platform for PDT in melanoma therapy. Phototherapy holds significant promise as an innovative approach for precision tumor treatment.

### 4.5 Microneedles for synergistic

Therapies Single-treatment approaches for melanoma often fail to achieve optimal outcomes due to limitations such as non-specific drug uptake, uncontrolled release, and systemic side effects (135). In recent years, combination therapy strategies have shown significant promise in addressing these challenges. Compared to monotherapy, microneedles used in combination therapies offer several advantages, including enhanced therapeutic efficacy, reduced drug doses, and decreased dosing frequency (136, 137). Additionally, microneedle-based combination therapies improve treatment convenience and minimize drug-induced damage to normal tissues. These features not only enhance patient compliance but also reduce off-target effects, making them particularly advantageous for melanoma treatment.

#### 4.5.1 Synergistic therapy of chemotherapy and photocatalysis

**Figure 8:**
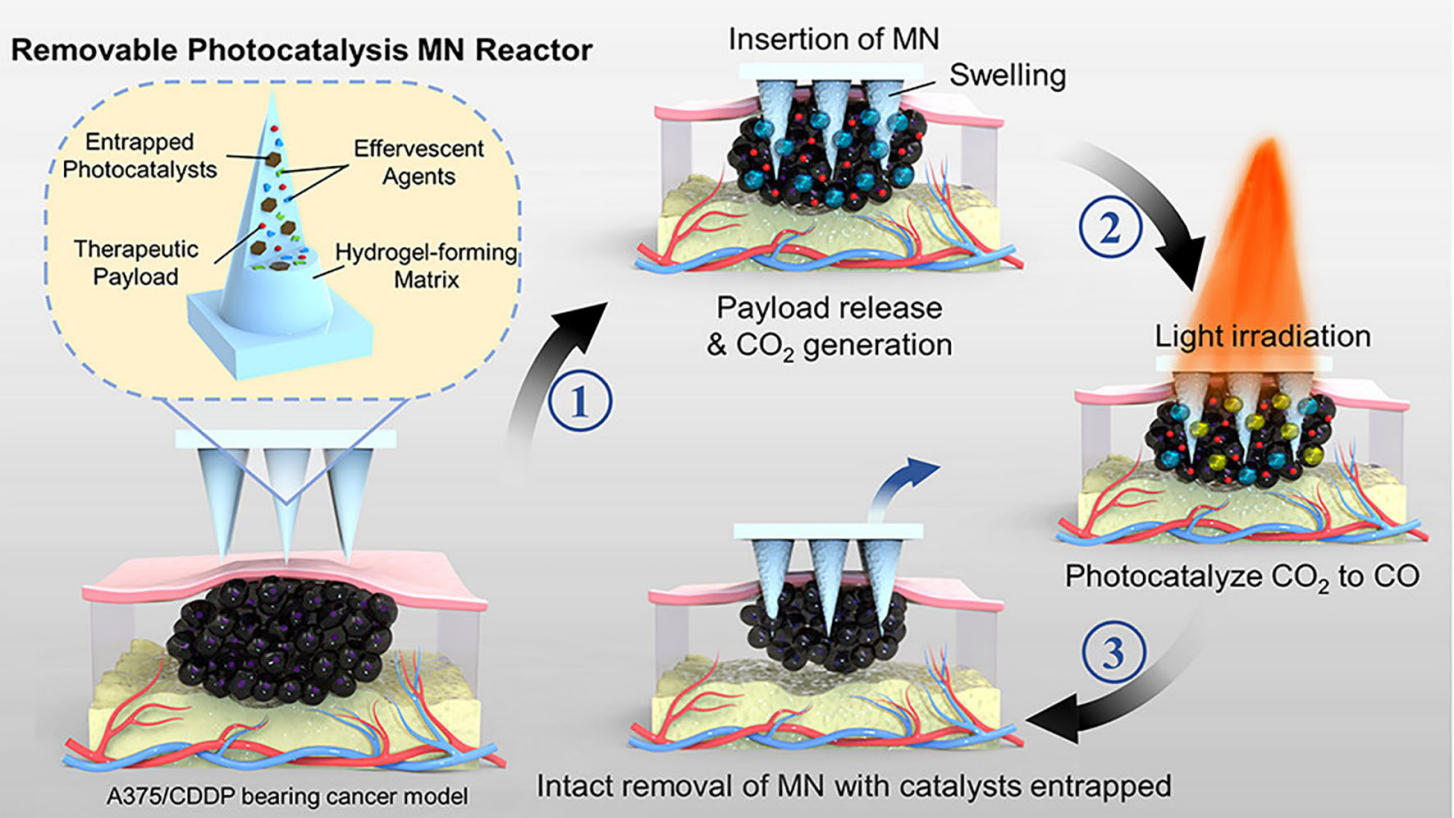
Schematic representation of a HMN platform for synergistic chemotherapy and photocatalytic therapy in the treatment of melanoma. Copyright permission from Yu et al. (105), *Nano Letters*, 2024.

The standalone application of chemotherapy is often limited by severe side effects and suboptimal efficacy. Combining chemotherapy with photocatalysis offers a promising strategy to address the limitations of single-treatment approaches. Recent studies have highlighted the therapeutic potential of carbon monoxide (CO), an endogenous signaling molecule involved in various physiological and pathological processes (138, 139). In cancer therapy, CO has been shown to enhance tumor cell sensitivity to chemotherapeutic drugs (140), suppress inflammation, and protect normal cells (141, 142). Photocatalytic CO2 reduction technology has emerged as a novel method for controlled CO delivery (143, 144). This approach converts CO2 into CO via a photocatalytic reaction, enabling precise and localized CO generation under light irradiation (145). However, current technologies face challenges, including the low concentration of CO2 at the tumor site and the toxicity of photocatalytic agents, necessitating the development of safer and more efficient delivery platforms. Addressing these challenges, Yu et al. (105) introduced a novel microneedle reactor that integrates photocatalytic CO2 reduction technology for precise, localized CO generation and delivery. This innovative platform enhances tumor responsiveness to chemotherapeutic agents and demonstrates significant inhibitory effects against melanoma (**Figure 8**). The microneedle reactor is fabricated from a copolymer of hydroxyethyl methacrylate (HEMA) and N-vinyl-2-pyrrolidone (NVP) and incorporates effervescent agents, therapeutic payloads, and photocatalysts. Effervescent agents, such as tartaric acid and sodium bicarbonate, react with body fluids to release CO2, while copper sulfide nanosheets act as the photocatalyst, converting CO2 into CO under 660 nm light irradiation. This process enables the localized and controlled delivery of CO to the tumor site. CO enhances the cytotoxic effects of chemotherapeutic drugs, such as cisplatin, by inhibiting DNA repair mechanisms in tumor cells. *In vivo* experiments demonstrated that the combination of microneedle-delivered CO and cisplatin treatment achieved superior tumor suppression in a melanoma mouse model. Tumor growth in the combination treatment group was only 1.2 times the initial tumor volume, significantly outperforming other treatment groups. Microneedle systems utilizing photocatalysis-generated CO enhance the sensitivity of chemotherapy by inhibiting DNA repair mechanisms and modulating tumor metabolism. The anti-inflammatory and cytoprotective properties of CO further contribute to mitigating chemotherapy-induced toxicity. Moreover, the microneedle platform enables the integration and controlled release of multiple therapeutic strategies. Despite its potential, this platform still faces challenges, including limited light penetration depth, difficulties in achieving precise control of CO concentrations, and potential risks associated with catalyst residues.

#### 4.5.2 Synergistic therapies for immunotherapy-related approaches

In melanoma immunotherapy, dendritic cell (DC)-based vaccines have demonstrated the potential to enhance immune responses (146). However, current DC engineering approaches face several limitations, including complex and costly ex vivo operations and poor lymph node homing ability, which reduce their anticancer efficacy (147, 148). To address these issues, Ye et al. (103) developed a melanin-mediated MeHA microneedle patch that integrates PTT with immunotherapy to strengthen antitumor immunity. This microneedle patch incorporates melanin nanoparticles and the immune adjuvant granulocyte-macrophage colony-stimulating factor (GM-CSF). Upon NIR light irradiation, the melanin nanoparticles absorb NIR light and convert it into heat, inducing local hyperthermia in tumor tissues. This process not only directly destroys tumor cells but also activates immune cells. Simultaneously, GM-CSF released from the microneedles promotes the activation of DCs, enabling them to recognize tumor antigens and stimulate T cells, thereby amplifying the antitumor immune response. The experimental results indicated that 87% of treated mice achieved complete tumor regression, and tumor growth was significantly slowed. This dual approach, leveraging both thermal effects and immune activation, effectively treated local tumors while simultaneously stimulating a systemic immune response, offering the potential to inhibit the growth of distant tumors. Microneedle systems for photothermal-immunotherapy offer promising therapeutic advantages. The melanin-mediated photothermal effect induces localized heat shock responses, facilitating the release of inflammatory factors and the recruitment of immune cells. Notably, GM-CSF and whole tumor lysates work synergistically to induce a robust systemic antitumor immune response.

**Figure 9:**
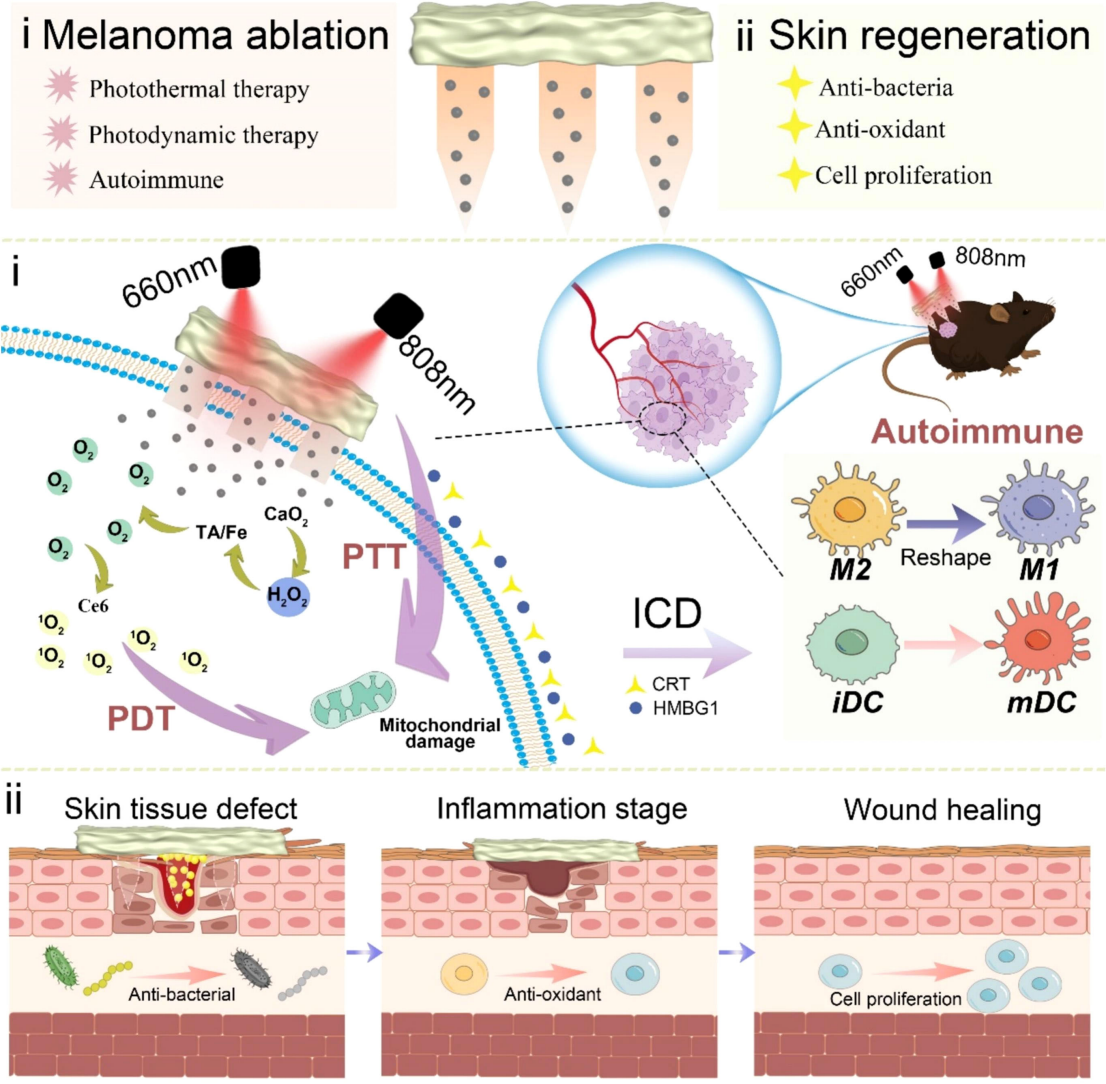
Schematic representation of a HMN platform for immune synergistic therapy in melanoma ablation and subsequent skin regeneration. Copyright permission from Zhang et al. (107), *International Journal of Biological Macromolecules*, 2024.

Traditional surgical treatments for melanoma face critical limitations, including the risk of tumor recurrence following excision and inadequate attention to skin regeneration during the treatment process. To address these challenges, Zhang et al. (107) developed a dual-functional microneedle hydrogel system based on hyaluronic acid, enabling synergistic tumor ablation and skin regeneration. This system features a nanomaterial encapsulated in the microneedle tips, comprising Carbon nanotubes@calcium peroxide@tannic acid-Fe/chlorin e6 (CCa@TF/Ce6). Under dual-laser irradiation, these materials generate ROS and heat through PTT and PDT. This process induces immunogenic cell death, promotes DC maturation, and remodels tumor-associated macrophages, ultimately activating autologous immunity to eliminate tumors. In a melanoma mouse model, the dual-laser irradiation group exhibited the strongest anti-melanoma effects, with significantly reduced tumor cell proliferation and a substantial decrease in tumor volume. Following tumor ablation, the microneedle base, composed of 3-aminophenylboronic acid-grafted HA-incorporated PVA hydrogel, facilitated tissue repair by reducing oxidative stress and promoting cell proliferation and migration. These findings underline the development of a therapeutic platform that integrates both antitumor efficacy and tissue regeneration capabilities (**Figure 9**). Nevertheless, key challenges remain to be addressed, including the validation of long-term efficacy, simplification of treatment protocols, and enhancement of system adaptability for personalized therapies, which are pivotal for advancing clinical translation. This synergistic therapy integrates PTT and PDT to locally generate high temperatures and ROS at the tumor site, effectively killing tumor cells. A notable advantage of this combination is its ability to remain effective under the hypoxic conditions commonly encountered in the tumor microenvironment. Furthermore, the immunogenic cell death induced by PTT and PDT enhances the immune system’s capacity to recognize and eliminate tumor cells. Despite these benefits, the immune-synergistic therapies discussed above also carry potential risks. While immune activation is essential for effective antitumor therapy, excessive immune responses may cause damage to normal tissues or trigger allergic reactions. Healthy skin tissues surrounding the tumor site are particularly vulnerable to immune-mediated inflammatory responses. Additionally, the localized thermal effects of PTT may harm adjacent normal tissues, especially when precise targeting of the treatment area is not achieved. Therefore, optimizing the synergistic ratios of therapeutic components and conducting comprehensive safety evaluations are critical to advancing these therapies toward clinical applications.

## 5 Conclusion and outlook

Melanoma, known for its high invasiveness, metastatic potential, and resistance to treatment, remains one of the most challenging cancers to manage. In recent years, hydrogel microneedle technology has emerged as a groundbreaking innovation in melanoma care, offering distinct advantages across the entire continuum—from early detection to treatment monitoring. Current research primarily focuses on three key areas: diagnosis, treatment, and materials, with significant progress achieved in each. From a diagnostic perspective, HMN technology has shown great promise in extracting and quantifying melanoma biomarkers, providing innovative, minimally invasive methods for early detection. In terms of therapeutic strategies, chemotherapy, immunotherapy, targeted therapy, phototherapy, and their multimodal synergistic combinations have opened new pathways for improving treatment outcomes. On the materials innovation front, developments range from the creation of foundational materials (e.g., hyaluronic acid, PVA, and GelMA) to intelligent responsive systems and optimized fabrication techniques, such as infrared-assisted manufacturing (149). In practical applications, HMNs enhance tumor sensitivity to therapeutic agents, reduce systemic drug toxicity, and improve drug targeting and bioavailability (150). Their minimally invasive nature enhances patient compliance, while the precise control of drug release opens new possibilities for personalized medicine (151). Furthermore, the integration of therapeutic and monitoring functions into a single platform enables real-time tracking and intervention, paving the way for more comprehensive and effective melanoma treatment strategies.

Despite these promising advancements, several significant challenges persist at both the technical and clinical translation stages. Technically, key challenges include variability in drug release control, insufficient mechanical strength of materials that compromise penetration efficiency, and limitations in drug-loading capacity. Additionally, issues related to the preparation process—such as the scaling up of complex drug delivery systems and achieving uniform dispersion of photocatalysts—need to be addressed to enable broader application. At the clinical translation level, major obstacles remain. Most current research relies heavily on preclinical mouse models, with limited validation in human clinical trials. The primary limitation stems from the inherent differences between animal models and humans, as human and mouse skin structures differ significantly in thickness, density, and cellular composition. Furthermore, the tumor growth cycle in mice is considerably shorter than the progression of melanoma in humans, and mouse models fail to fully replicate the complexity of the human immune microenvironment. Discrepancies in body weight further complicate the conversion of drug dosages between species, creating substantial uncertainty. These fundamental differences intensify the challenges of translating therapeutic effects from preclinical models to human applications. In drug delivery systems, optimizing drug-loading capacity to meet human physiological demands is essential. Additionally, dosing frequency and interval adjustments must be tailored to human physiological characteristics. Safety evaluation also presents significant challenges: the intensity and patterns of immune responses in humans may differ markedly from those in mice, underscoring the urgent need for a more robust adverse reaction monitoring framework. Moreover, variability in individual immune responses adds complexity to tailoring therapies for personalized treatment needs. Long-term safety concerns, including the potential leakage of catalysts and risks associated with nanomaterials, further contribute to uncertainty regarding the clinical applicability of hydrogel microneedle technology.

Looking ahead, research efforts should focus on four key areas. First, in material and technology optimization, there is a need to explore novel intelligent responsive materials and simplify preparation processes. Second, in therapeutic strategy innovation, the focus should be on developing integrated diagnosis and treatment platforms, expanding the range of detectable biomarkers, and optimizing multimodal combination therapies. Third, in clinical translation, it is crucial to strengthen clinical trials, accumulate more *in vivo* data, and establish standardized safety evaluation systems. Additionally, while most microneedle studies primarily target local primary tumors, metastatic melanoma has been largely overlooked, therefore, future research should focus more on metastatic melanoma. Finally, in interdisciplinary integration, proactive collaboration across multiple disciplines is essential to expedite the translation of research findings into clinical applications. In conclusion, HMN technology holds great potential for melanoma treatment. Future research should focus on overcoming existing technical challenges and accelerating clinical translation to deliver more precise and effective therapies for melanoma patients.

## Glossary

5-FU: 5-fluorouracil
α-MSH: α-melanocyte-stimulating hormone
alloc-DOX: allyloxycarbonyl-doxorubicin
AZA: azelaic acid
Ca²⁺: calcium ion
CCa@TF/Ce6: Carbon nanotubes@calcium peroxide@tannic acid-Fe/chlorin e6
CO: Carbon monoxide
CpG-ODNs: cytosine-phosphate-guanine oligodeoxynucleotides
CS: chitosan
Cu²⁺: copper ions
CZCH: Cu2+-doped porous zeolitic imidazolate framework incorporated with catalase
DA: dopamine
DexMA: dextran methacrylate
DOX: doxorubicin
EAA: 3-O-ethyl ascorbic acid
GelMA: gelatin methacryloyl
GelMA-β-CD: gelatin methacryloyl and β-cyclodextrin
GM-CSF: granulocyte-macrophage colony-stimulating factor
GSH: glutathione
HA: hyaluronic acid
HAT: hyaluronic acid-tyramine
HEMA: hydroxyethyl methacrylate
HMNs: hydrogel microneedles
ISF: interstitial fluid
LAP: laponite
MAT: matrine
MeHA: methacrylated hyaluronic acid
MMPs: magnetic microparticles
MNs: Microneedles
MOF: metal–organic framework
MSC: mesenchymal stem cell
NDV: Newcastle disease virus
NIR: near-infrared
NVP: N-vinyl-2-pyrrolidone
OVA: ovalbumin
Pd: palladium
PDMS: polydimethylsiloxane
PDT: photodynamic therapy
PEG: poly(ethylene glycol)
PEGdiacid: poly(ethylene glycol) diacid
P-gp: P-glycoprotein
PMPs: polystyrene microparticles
PMVE/MA: poly(methyl vinyl ether-co-maleic acid)
PTT: photothermal therapy
PVA: Poly(vinyl alcohol)
PVP: polyvinylpyrrolidone
ROS: reactive oxygen species
SA: sodium alginate
SF: silk fibroin
TDDS: Transdermal Drug Delivery Systems
TME: tumor microenvironment
TMPyP: tetrakis(1-methyl-4-pyridinio) porphyrin
TNSs: TiO_2_ nanosheets
Tra: Trametinib
